# Correlated MRI and Ultramicroscopy (MR-UM) of Brain Tumors Reveals Vast Heterogeneity of Tumor Infiltration and Neoangiogenesis in Preclinical Models and Human Disease

**DOI:** 10.3389/fnins.2018.01004

**Published:** 2019-01-10

**Authors:** Michael O. Breckwoldt, Julia Bode, Felix Sahm, Thomas Krüwel, Gergely Solecki, Artur Hahn, Peter Wirthschaft, Anna S. Berghoff, Maximilian Haas, Varun Venkataramani, Andreas von Deimling, Wolfgang Wick, Christel Herold-Mende, Sabine Heiland, Michael Platten, Martin Bendszus, Felix T. Kurz, Frank Winkler, Björn Tews

**Affiliations:** ^1^Neuroradiology Department, Heidelberg University Hospital, Heidelberg, Germany; ^2^Clinical Cooperation Unit Neuroimmunology and Brain Tumor Immunology, German Cancer Research Center (DKFZ), Heidelberg, Germany; ^3^Schaller Research Group at the University of Heidelberg and the German Cancer Research Center (DKFZ), Molecular Mechanisms of Tumor Invasion, Heidelberg, Germany; ^4^Department of Neuropathology, Heidelberg University Hospital, Heidelberg, Germany; ^5^Clinical Cooperation Unit Neuropathology, German Cancer Research Center (DKFZ), Heidelberg, Germany; ^6^Clinical Cooperation Unit Neurooncology, German Cancer Consortium (DKTK) Within the DKFZ, Heidelberg, Germany; ^7^Institute for Anatomy and Cell Biology, Heidelberg University, Heidelberg, Germany; ^8^Neurology Clinic and National Center for Tumor Diseases, Heidelberg University Hospital, Heidelberg, Germany; ^9^Division of Experimental Neurosurgery, Department of Neurosurgery, University of Heidelberg, Heidelberg, Germany; ^10^Department of Neurology, University Medical Center Mannheim, University of Heidelberg, Mannheim, Germany

**Keywords:** magnetic resonance microscopy, ultramicroscopy, brain tumor models, tumor invasion, brain clearing, glioblastoma

## Abstract

Diffuse tumor infiltration into the adjacent parenchyma is an effective dissemination mechanism of brain tumors. We have previously developed correlated high field magnetic resonance imaging and ultramicroscopy (MR-UM) to study neonangiogenesis in a glioma model. In the present study we used MR-UM to investigate tumor infiltration and neoangiogenesis in a translational approach. We compare infiltration and neoangiogenesis patterns in four brain tumor models and the human disease: whereas the U87MG glioma model resembles brain metastases with an encapsulated growth and extensive neoangiogenesis, S24 experimental gliomas mimic *IDH1* wildtype glioblastomas, exhibiting infiltration into the adjacent parenchyma and along white matter tracts to the contralateral hemisphere. MR-UM resolves tumor infiltration and neoangiogenesis longitudinally based on the expression of fluorescent proteins, intravital dyes or endogenous contrasts. Our study demonstrates the huge morphological diversity of brain tumor models regarding their infiltrative and neoangiogenic capacities and further establishes MR-UM as a platform for translational neuroimaging.

## Introduction

Gliomas with their most malignant entity glioblastoma are highly malignant brain tumors with poor prognosis and a median survival of ~15 months (Wen and Kesari, 2008). Gliomas are characterized by high cellular proliferation rates, induction of neoangiogenesis and a diffuse and infiltrative growth into the adjacent healthy brain (Furnari et al., 2007; Carmeliet and Jain, 2011). This infiltrative nature prevents more effective therapy because surgical resection and radiotherapy regimens are locally confined (Sahm et al., 2012; Osswald et al., 2015). Glioma invasion is also a challenge for treatment monitoring by magnetic resonance imaging (MRI) due to insufficient resolution (~0.5 mm for most clinical scanners) and a lack of specific MRI sequences that can detect infiltrating glioma cells (Hyare et al., 2017; Smits and van den Bent, 2017).

So far, only very laboursome methods such as cryo-imaging that acquired thousands of physically tiled images allowed to visualize whole organs while also providing the resolution of single fluorescently labeled cells (Wilson et al., 2008; Qutaish et al., 2012). Yet, these methods lack dynamic and longitudinal imaging aspects important for monitoring tumor development and treatment response.

We have previously developed a correlated magnetic resonance imaging and ultramicroscopy approach (dubbed “MR-UM”) to monitor glioma angiogenesis at single vessel resolution (Breckwoldt et al., 2016). Using an extended approach to monitor cellular infiltration and angiogenesis site by site we now map these key parameters by MRI and dual color ultramicroscopy in glioma models and compare our findings to an established model of brain metastases (Holland, 2001; Zhu et al., 2014; Osswald et al., 2015). We further use human specimen of brain metastases and resected gliomas (*IDH1* wildtype glioblastoma and *IDH1* mutant oligodendroglioma) to further advance our technique to the clinical arena.

For our analysis, we compared the conventional *IDH1* wildtype, *PTEN* mutant, *p14ARF*, and *p16* deleted U87MG model (Ishii et al., 1999; Chen et al., 2012) that has a PI3K/Akt pathway up-regulation as a result of high Akt expression (Koul et al., 2006; Radaelli et al., 2009) with a PDGFß-driven, *Ink4*-*Arf*-deleted, *Pten* wildtype RCAS/t-va model replication-Competent Avian Sarcoma-leukosis virus long-terminal repeat with splice acceptor (RCAS)-tumor virus A (TVA) gene delivery system (Hambardzumyan et al., 2009), the metastatic melanoma model (A2058) (Sherwin et al., 1979), and the *IDH1* wildtype (WT) S24 model kept under serum-free, stem-like conditions (Osswald et al., 2015).

We used fluorescent protein expression of tumor cells to map tumor infiltration and intravital dye labeling to assess angiogenesis by ultramicroscopy. MRI was performed longitudinally during tumor growth using advanced MRI techniques. MR-UM distinguished different infiltration patterns of single tumor cells and patterns of angiogenesis in mouse and human brain tumors. Our findings are that the conventional U87MG gliomas resemble human brain metastasis regarding their spherical growth and extensive neoangiogenesis, whereas the recently described S24 gliomas phenocopy human glioblastomas showing extensive infiltration into the adjacent parenchyma and along white matter tracts to the contralateral hemisphere.

## Methods

### U87MG Glioma Model

The human glioma cell line U87MG was obtained from LGC Standards (Wesel, Germany) and cultured in Dulbecco's modified Eagle's medium (DMEM) containing 10% FBS, 100 U/ml penicillin and 100 μg/ml streptomycin (all from Sigma-Aldrich, Taufkirchen, Germany). Six to eight week old male NMRI nude mice were injected stereotactically with 50.000 U87MG-tdTomato cells in 2 μl PBS. Implantation was performed into the right hemisphere, 1 mm rostral and 2 mm lateral from the Bregma at a depth of 500 μm (*n* = 5 mice, Charles River, Sulzfeld, Germany) using a Hamilton syringe, driven by a fine step motor. Animals were anesthetized with ketamine/xylazine and unresponsive to stimuli during the intracranial injection. MRI was performed on day 16 and 28 post tumor cell implantation.

### RCAS/t-va Model

DF-1 cells, a chicken fibroblast cell line, was obtained from LGC Standards and cultured in DMEM containing 10% FBS, 100 U/ml penicillin and 100 μg/ml streptomycin (all from Sigma-Aldrich) at 39°C. To model astrocytoma tumorigenesis, replication-competent avian leukosis virus with splice acceptor (RCAS) viral vectors containing PDGF gene and *Discosoma* sp. red fluorescent protein (DsRed) were used for the transfection of cells. Nestin-Tv-a mice were anesthetized at postnatal days 5 to 8 with isoflurane (2%) and 40.000 DF-1 cells in a total volume of 1 μl PBS were injected into the brain. High grade gliomas developed 4.5 to 10 weeks after injection, albeit with low frequency (30%). Animals that developed hydrocephalus were sacrificed and excluded from the study (*n* = 4 mice were included in the study). MRI was performed weekly starting 5 weeks after inoculation.

### S24 Model

The S24 cell line was derived as a primary glioblastoma culture from an IDH1 wildtype glioblastoma (Lemke et al., 2012). For the S24 glioma model, 50.000 S24 cells in 2 μl total volume were injected into the right basal ganglia (coordinates: 1 mm rostral and 2 mm lateral from the Bregma at a depth of 2 mm) in 8–10 week old male NMRI nude mice (Charles River, n = 6 mice). Cells were cultivated under serum-free conditions in DMEM-F12 as sphere cultures supplemented with 2% B-27 (Thermo Fisher Scientific Inc., Waltham, MA, USA), 5 μg/ml human insulin (Sigma-Aldrich), 12.8 ng/ml heparin (Sigma-Aldrich), 0.4 ng/ml EGF (R&D Systems Inc., Minneapolis, MN, USA), and 0.4 ng/ml FGF (Thermo Fisher Scientific). MRI was performed on day 27, 48, 70, 84, 91 after tumor cell implantation.

### A2058 Brain Metastasis Model

The amelanotic melanoma cell line A2058 (obtained from the ATCC-CRL-11147, 07/2011) was cultivated under standard condition in DMEM media containing 10% FBS, 100 U/ml penicillin and 100 μg/ml streptomycin (all from Sigma-Aldrich). Cells underwent 4 *in vivo* passages to increase the brain metastatic potential as previously described (Osswald et al., 2016). A cytoplasmic RFP-tdTomato, LeGo-T2 vector (kind gift from A. Trumpp, German Cancer Research Center) was used for transfection and generation of a fluorescent cell line. 500.000 cells in 100 μl PBS were injected in the left ventricle of the mouse (*n* = 5 mice). Animals were monitored weekly by MRI starting 2 weeks after intracardial tumor cell injection.

All cell lines were routinely tested for contamination by the multiplex cell contamination test (Schmitt and Pawlita, 2009). The test detects 28 potential cell culture contamination such as mycoplasma (14 different species), Epstein-Barr Virus and 14 possible cross contaminations with cells of human, mouse etc origin. Animal experiments were approved by the regional animal welfare committee (permit numbers: G8/14 and G189/12, Regierungspräsidium Karlsruhe, Germany).

### Human Brain Metastasis Samples

Two human brain metastases samples from a lung adenocarcinoma were obtained during routine pathological autopsy and written informed consent was obtained. Brain tissue blocks (~2 × 2 × 2 cm) included metastatic sites from the cerebellum and cortex. Tissue blocks were subjected to high resolution *ex vivo* MRI and subsequent clearing and ultramicroscopy. Additional tissue sections were stained for H&E, CD31, and cytokeratin AE1/AE3 using routine histopathological processing.

### Human Glioma Samples

One glioblastoma and one oligodendroglioma tissue block were obtained during surgical resection (~1 × 1 × 1 cm; glioblastoma: *IDH1* wildtype; oligodendroglioma: *IDH1* R132H mutation, 1p19q co-deletion). Use of patient material was approved by the Institutional Review Board at the Medical Faculty Heidelberg and written informed consent was obtained from all patients included in the study (ethics approval: 005/2003). Tissue was immediately transferred to 4% PFA and tissue blocks were subjected to high resolution *ex vivo* MRI and subsequent clearing and ultramicroscopy. MR images of the respective patient was acquired 1 day prior to surgery using routine clinical MRI sequences on a 3 Tesla MRI system (Siemens, Erlangen, Germany). Additional tissue sections were stained for H&E, CD31, and IDH1R132H using routine histopathological processing.

### MR Imaging

MR imaging was performed on a 9.4 Tesla horizontal bore small animal NMR scanner (BioSpec 94/20 USR, Bruker BioSpin GmbH, Ettlingen, Germany) and performed as previously described (Breckwoldt et al., 2016). Briefly, a standard RARE T2-w and T1-w post-Gd-contrast sequence was used to monitor tumor volume (T2-w parameters: 2D sequence, TE: 33 ms, TR: 2,500 ms, flip angle: 90°, acquisition matrix: 200 x 150, number of averages: 2, slice thickness: 700 μm duration: 2 min 53 s; T1-w parameters: TE: 6 ms, 1,000 TR: ms, flip angle: 90°, acquisition matrix: 256 x 256, number of averages: 2, slice thickness: 500 μm, duration: 5 min). We also used a 3D T2-w sequence: TE 33 ms; TR: 1,800 ms; flip angle: 90°; acquisition matrix: 200 × 200; number of averages: 1; in plane resolution: 100 μm; duration: 10 min 48 s. The T1-w parameters were as follows: 3D sequence: TE: 1.9 ms; TR: 5 ms; flip angle: 60°; acquisition matrix: 128 × 128; number of averages: 4; in-plane resolution: 156 μm; duration: 5 min 28 s. To assess the tumor vasculature we used a T2^*^-w, gradient echo sequence (Park et al., 2008) and acquired pre- and post-contrast scans (3D sequence, 80 μm isotropic resolution, TE: 18 ms; TR: 50 ms; flip angle: 12°; number of averages: 1, acquisition matrix: 400 x 400, duration: 15 min 40 s). Pre-contrast images were used to differentiate susceptibility artifacts caused e.g., by tumor microbleedings from vessel signals that were only detectable after contrast administration. Dynamic contrast enhanced imaging (DCE) was used to assess vascular permeability (K_trans_) (TE: 1.8 ms; TR: 16 ms; flip angle: 10°; slice thickness: 700 μm, acquisition matrix: 66 × 128, 3 slices acquired, number of averages: 1, 300 repetitions; 700 μm in plane resolution; duration: 6 min, time resolution 2 s). 0.2 mmol/kg Gd-DTPA-BMA (Omniscan, Nycomed, Ismaningen, Germany) was administered by tail vein injection for DCE and post-contrast scans. For MR imaging, animals were anesthetized with 1–2% isoflurane and kept on a heating pad. Respiration was monitored externally with a surface pad controlled by an in house developed LabView program (National Instruments Corporation, Austin, USA).

### Image Processing and Analysis of MRI Data

The tumor volume was quantified in Osirix software (V.4.12, Pixmeo, Geneva, Switzerland) by manually selecting ROIs around the tumor outline on T2-w images. Segmentation of vessels was performed on T2^*^w images based on the typical tubular hypointense structure using AMIRA (Thermo Fisher Scientific, Hillsboro, USA). Quantification of DCE timeseries was performed in FIJI by ROI analysis of the entire tumor area. Intensity values were measured and processed in Microsoft Excel (Microsoft, USA). Signals were normalized to the time period before contrast administration (S/S_0_) and displayed as “permeability” (arbitrary units, a.u.).

### Labeling of the Microvasculature

For labeling of the microvasculature, animals were injected with fluorescent lectins that bind to N-acetyl-β-D-glucosamine oligomers of endothelial cells (Wälchli et al., 2015; Breckwoldt et al., 2016). Isolectin-FITC or lectin texas-red (12 mg/kg, Sigma-Aldrich or Vector Laboratories, Burlingame, CA USA) was injected iv in 100 μl PBS after the last MRI. Animals were sacrificed 5 min after lectin injection by a ketamine/xylazine overdose and transcardially perfused with 20 ml PBS followed by 20 ml 4% PFA (Histofix, Carl Roth, Karlsruhe, Germany).

### Fixation and Clearing of Mouse and Human Samples

Mouse brains were fixed after perfusion with 4% PFA for 24 h and stored in PBS at 4°C in the dark. For UM-analysis whole brains were optically cleared using the FluoClearBABB protocol (Schwarz et al., 2015; Alves et al., 2016; Breckwoldt et al., 2016). The protocol is based on benzyl alcohol/benzyl benzoate clearing in combination with a basic pH. For the dehydration of brains analytical grade alcohol (t-butanol, Sigma-Aldrich) was diluted with double-distilled water. Brains were dehydrated using t-butanols ranging from 30 to 100%. The clearing solution BABB was prepared by mixing benzyl alcohol (Merck, analytical grade) and benzyl benzoate (Sigma-Aldrich, “purissimum p.A.” grade) in a 1:2 volume ratio. The pH levels of dehydration and clearing solutions were adjusted using an InLab Science electrode suited for organic solvents (Mettler-Toledo, Columbus, Ohio, United States). pH levels were adjusted with triethylamine (Sigma-Aldrich). BABB cleared samples were stable over time and showed no apparent decrease in fluorescence signal. Samples were kept in BABB at 4°C in the dark. The stability allowed re-imaging of the sample over time. Bleaching of samples was also not a major issue.

For clearing of human samples, we used the iDISCO+ protocol (Renier et al., 2014). The working distance of the objective is 1 cm. Therefore, we needed to trim human tissue blocks to the appropriate size (~0.5–1cm^3^) in order to place the sample under the objective.

### Acquisition and Analysis of Ultramicroscopy Data Sets

Cleared brains were scanned with a light sheet microscope (LaVision BioTec GmbH, Bielefeld, Germany). We used a magnification of 1.0x and 2.0x with a 2x objective lens and a white light laser (SuperK EXTREME 80 mHz VIS; NKT Photonics, Cologne, Germany). For lectin stained vessels, the following filters were used: lectin-FITC, excitation 470/40 nm; emission 525/50 nm; lectin-texas red excitation 545/25; emission 585/40. Z-stacks of 5 μm step size and a total range of 1,500–2,000 μm for transversal measurement of whole brains were acquired. The resolution of our light sheet microscope setup is 6 × 6 μm with a lightsheet thickness of 2 μm. In comparison to MRI this is at least a 13-fold higher resolution (our T2^*^ sequence has an isotropic resolution of 80 μm). The method allows to image single tumor cells invading into the adjacent parenchyma or microvessels that are ~5 μm in diameter. Images were exported as tagged image file (tif) and further processed in ImageJ package FIJI, version 1.49 (http://fiji.sc/Fiji). Segmentation of fluorescent tumor cells or the microvasculature in the ultramicroscopy datasets was performed with Ilastik (Haubold et al., 2016). Segmentation was done in a supervised, semiautomated fashion.

### Histology

After fixation, S24-tdTomato tumor-bearing brains were coronally cut into 100 μm sections with a vibratome (Sigmann Elektronik, Hüffenhardt, Germany). Sections were permeabilized with 0.2% TX100 for 3 h, stained with DAPI (Vectorshield, Vector Laboratories) and mounted on coverslips. Images were acquired on a confocal laser-scanning microscope (Leica SP8, Leica, Germany) using a immersion oil objective with a 63-fold magnification (numeric aperture = 1.4). Images were acquired as tile scans at a pixel size of 141 and 300-nm z steps within a field of view of 655.92 × 920.07 μm.

### Statistical Analysis

Data is shown as mean ± S.E.M in graphs or mean ± S.D. with 95% confidence interval (C.I.) in results section. Statistical analyses were performed in PRISM (GraphPad La Jolla, USA). Two-tailed student's *t*-tests were used to compare two groups. One-way ANOVA with Bonferroni's *post-hoc* testing was used for multiple comparisons. *p*-values < 0.05 were considered significant, ^*^p < 0.05; ^**^p < 0.01; ^***^p < 0.001.

## Results

### U87MG Mouse Tumors Show Intratumoral Heterogeneity

**Figure 1:**
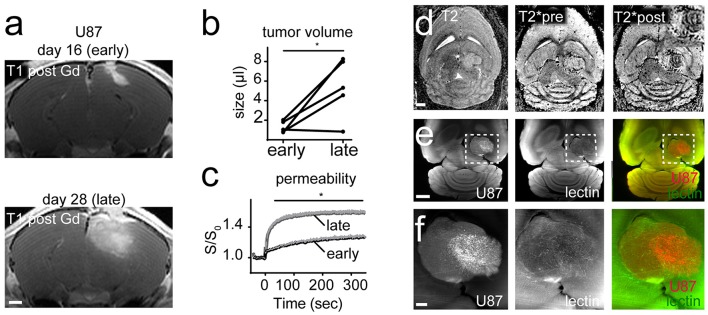
MR-UM of U87MG shows tumor heterogeneity. T1-w images post Gd-contrast application of U87MG tumors 16 days (early) and 28 days (late) after cortical tumor inoculation (*n* = 5 mice; **A**). Tumor volume quantification **(B)**. Dynamic contrast enhanced imaging shows vascular permeability over time **(C)**. T2-w, T2^*^ pre and after Gd-contrast application **(D)**. Inset in the upper right corner shows that only the lateral tumor compartment exhibits hypointense tubular vessels. Ultramicroscopy performed after tissue clearing of DS-red labeled U87MG tumor cells and lectin-FITC labeled vessels **(E)**. Magnified images depict two compartments of the bulk tumor **(F)**. Images in **(F)** are maximum intensity projections. Scale bars are 1 mm in **(A,D,E)**, and 100 μm in **(F)**. ^*^p < 0.05.

To assess the capability to perform dual color MR-UM we employed the U87MG tumor model to visualize tumor cells (U87MG-tdTomato) and the microvasculature (lectin-FITC) concomitantly. We monitored U87MG tumors longitudinally by MRI and imaged mice at day 16 and 28 after tumor implantation into the right cortex. MRI after gadolinium (Gd)-contrast administration showed the expected rapid growth with blood-brain barrier disruption (BBB-D, tumor volume day 16: 1.3 μl ± 0.58 vs. day 28: 5.4 μl ± 3.0; CI: 0.92 to 7.2; *p* = 0.02; Figures 1A,B). Dynamic contrast-enhanced imaging showed a progressive increase of vascular permeability during the course of tumor progression, suggestive of ongoing neoangiogenesis (*p* < 0.05, Figure 1C). Tumor vessels were also discernible by T2^*^-w imaging following Gd-contrast administration as hypointense tubular structures in parts of the tumor core (Figure 1D).

After the completion of MR measurements animals were injected with lectin-FITC for microvasculature labeling and subjected to brain clearing using the FluoClearBABB protocol (Schwarz et al., 2015). Unexpectedly, ultramicroscopy showed intratumoral heterogeneity with areas of increased tumor cell density and pathological vascularization in some parts of the tumor, whereas adjacent tumorous areas exhibited markedly less tumor cells and only few neovessels (Figures 1E,F). As expected U87MG tumors grew bulky with a clear separation of the tumor from the adjacent healthy parenchyma (Figure 1F).

### RCAS Tumors Are Highly Angiogenic and Show Multiple Intratumoral Microbleedings

**Figure 2:**
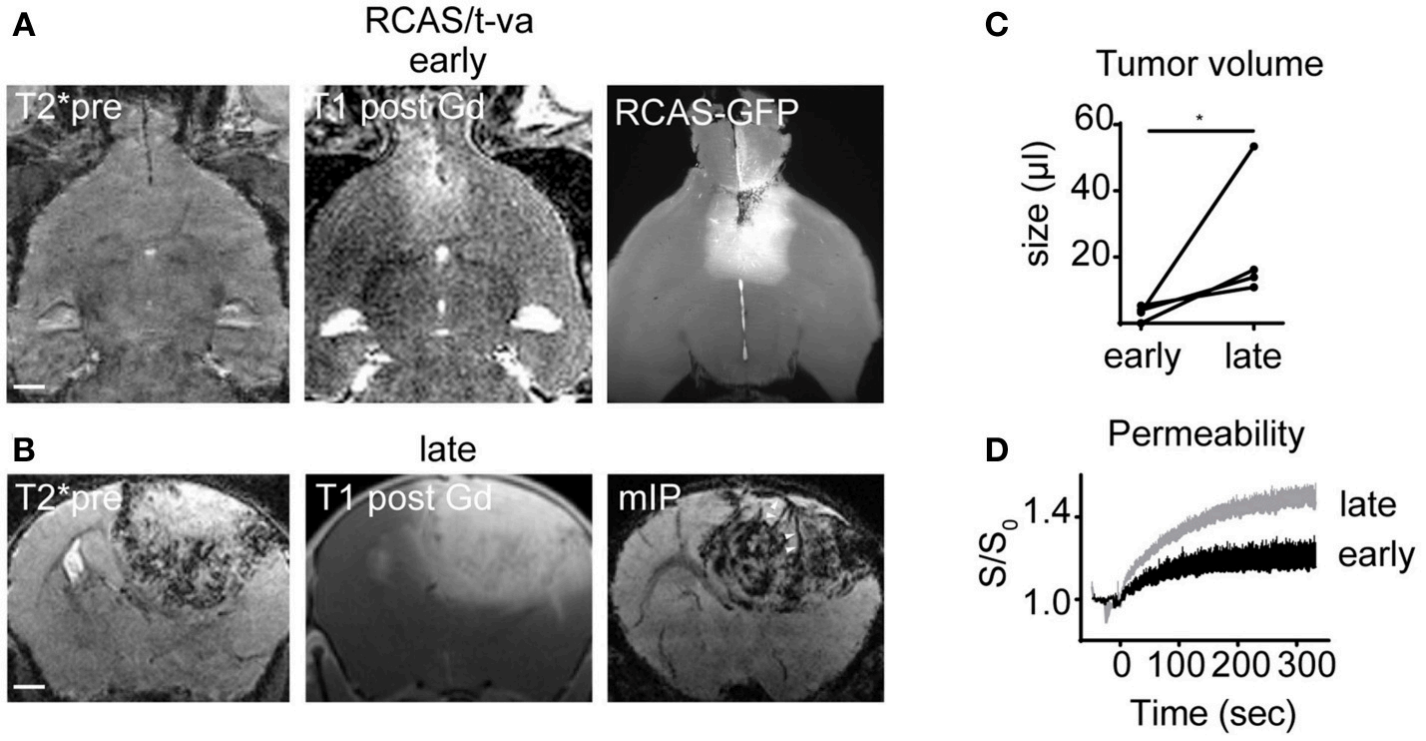
MR-UM of RCAS/t-va tumors. Representative T2^*^, T1 post and ultramicroscopy image at an early tumor stage (*n* = 4 mice; **A**). Lower row shows T2^*^, T1 post and minimum intensity projection (mIP) at a late tumor stage **(B)**. Quantification of tumor volume **(C)** and dynamic contrast enhanced imaging **(D)**. Arrowheads in mIP indicate tumor vessels. Scale bars in **(A,B)** are 1 mm. ^*^p < 0.05.

To further dissect infiltration patterns in a more recently described autochthonous glioma model we used the RCAS/t-va system. RCAS tumors grew initially slow and were only visible by MRI at 3 to 4 weeks after virus transduction (Figure 2A). At this early timepoint no microbleedings were detectable on T2^*^. However, upon initial tumor detection by MRI, tumors grew rapidly with massive angiogenesis, blood-brain barrier disruption and the development of intratumoral microbleedings at the tumor-brain parenchyma border as visualized by T2^*^ and DCE imaging (early tumor volume: 3.1 μl ± 2.2 vs late tumor volume: 23.5 μl ± 20.0; CI: −4.1 to 45; *p* < 0.05; Figures 2B–D). Contrary to our expectation we did not find apparent infiltrative growth of RCAS/t-va tumors on ultramicroscopy but rather a clearly defined tumor-parenchyma border (Figure 2A).

### S24 Tumors Grow Slowly Over Time and Diffusely Infiltrate Into the Adjacent Brain Using White Matter Tracts as Guide Structures

**Figure 3:**
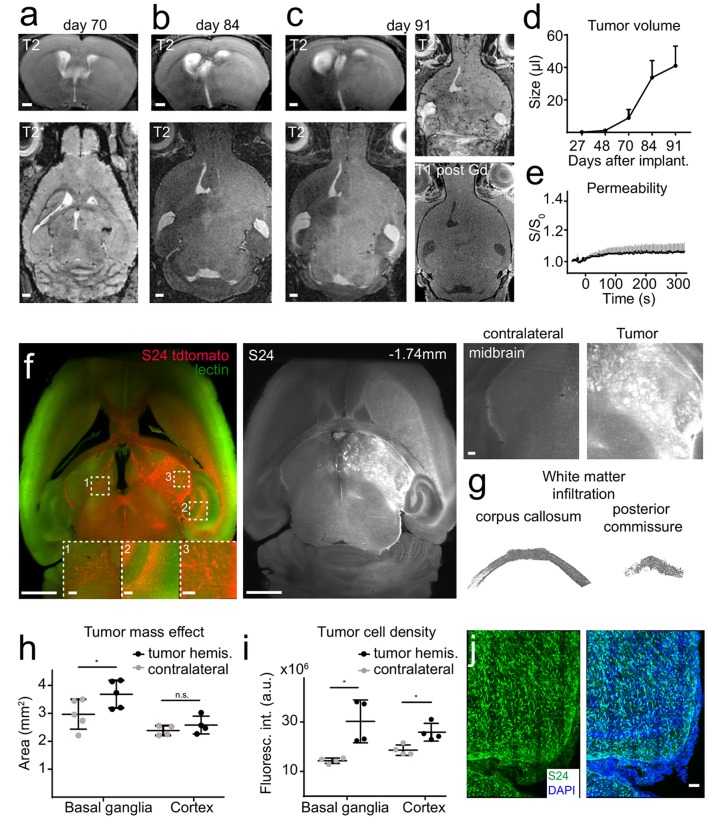
S24 tumors show a diffuse infiltration in the adjacent brain parenchyma. Longitudinal T2w images of S24 tumors at day 70 **(A)**, 84 **(B)**, and 91 **(C)** after tumor cell implantation into the midbrain (*n* = 6 mice). S24 tumors do not show intratumoral susceptibility changes nor Gd-contrast enhancement **(C)**. Quantification of tumor size based on T2 hyperintense areas **(D)** and vascular permeability as assessed by DCE MRI **(E)**. Ultramicroscopy image of S24 tdTomato cells labeled with lectin FITC **(F)**. S24 tumor spread occurs throughout the injected hemisphere and to the contralateral site. Magnified images of the midbrain, hippocampus, and contralateral hemisphere illustrate tumor cell invasion. Segmentation of single S24 tumor cells (depicted in gray) in the corpus callosum and in the posterior commissure **(G)**. Quantification of the total area **(H)** and fluorescence intensity **(I)** of the basal ganglia and the cortex illustrates the mass effect and volume increase caused by the tumor. Confocal micrograph (recorded as composite image, tile scan) of an S24 tumor section **(J)**. Scale bars are 1 mm in **(A–C)**, 50 μm in **(J)** and 1 mm in **(F)** (100 μm in magnified images). ^*^p < 0.05.

S24 gliomas are patient derived, glioma stem-like cells that have been recently described by our group (Osswald et al., 2015). S24 xenografts form large networks of interconnected tumor cells that use tumor microtubes for intercellular signaling and equilibration of the network, thus mediating resistance against radiochemotherapy (Jung et al., 2017; Weil et al., 2017). We implanted S24 tumors in the midbrain and performed MRI measurements longitudinally 4, 6, 10, 12, and 13 weeks after tumor implantation. Tumors were detected by MRI around week 10 and behaved markedly different than U87MG or RCAS/t-va tumors. MRI changes were only apparent on T2w imaging with subtle T2 hyperintensity and a progressing mass effect, whereas there was no obvious neoangiogenesis nor intratumoral microbleedings and only marginal blood-brain barrier disruption, (Figures 3A–E). After brain clearing, however, the full extent of diffuse tumor infiltration became apparent. Defined tumor-brain parenchyma borders were missing and infiltration followed white matter structures, such as the corpus callosum or the posterior commissure (Figures 3F,G, Supplementary Movies 1–3). There was also profound infiltration into the basal ganglia and to the contralateral hemisphere, showing a mass effect and increased fluorescence intensity caused by tdTomato expressing S24 cells in the basal ganglia and cortex (Figures 3H,I). To further visualize tumor microtubes and interconnected tumor cells, we performed confocal microscopy of S24 vibratome sections which confirmed the spread of tdTomato-labeled S24 cells throughout the entire hemisphere (Figure 3J).

### The Melanoma Brain Metastasis Model A2058 Shows Rapid, Encapsulated Growth Without Infiltration

To further complete the picture of brain tumor lesions, we performed experiments in a mouse melanoma metastasis model. After intracardial injection of A2058 melanoma cells, metastases developed in a seemingly random fashion mainly in cerebral hemispheres and at the basal entry points of the large brain supplying arterial vessels (Supplementary Figures 1A,B). Metastases grew agressively within 1 week after the first detection by MRI with severe BBB-D and neovessel formation, as depicted by T2^*^ imaging and ultramicroscopy after lectin-FITC injection (early tumor volume: 1.3 μl ± 1.2 vs. late tumor volume: 18.3 μl ± 15.3; CI: −1.4 to 35.3; *p* = 0.03; Supplementary Figures 1C–E). The growth pattern was restricted to an encapsulated form without apparent infiltration.

### Human Brain Metastasis Show an Encapsulated Growth Pattern

**Figure 4:**
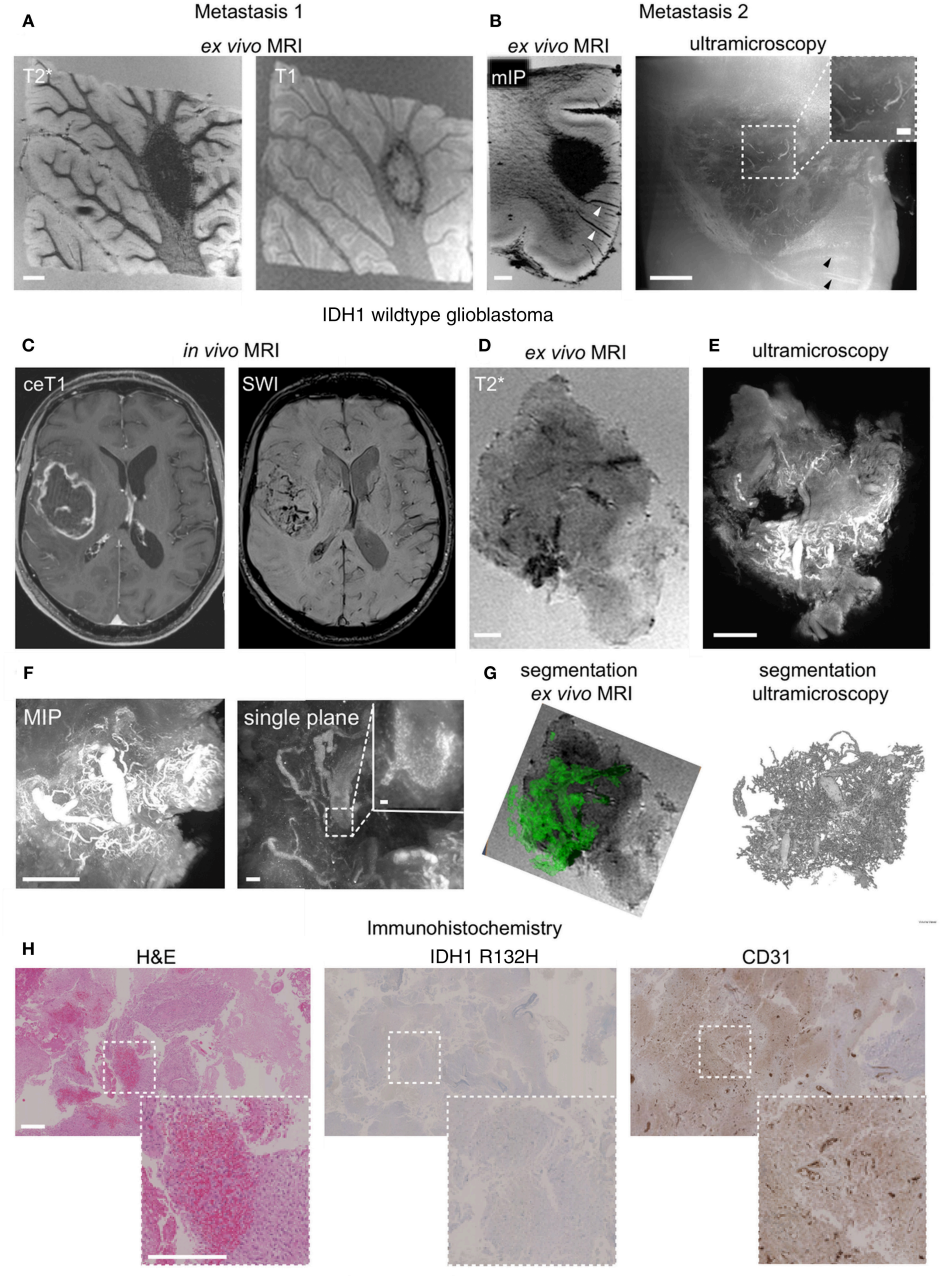
MR-UM of human brain metastasis and glioblastoma. T2^*^ and T1-w images of a human brain metastasis in the cerebellar white matter **(A)**. Depiction of a subcortical metastasis by T2^*^, shown as minimum intensity projection, mIP to illustrate hypointense tumor draining vessels (arrowhead) and ultramicroscopy **(B)**. *In vivo* MRI of human glioblastoma (*IDH1* wildtype) patient and after specimen resection **(C,D)**. Ultramicroscopy shows the Tortuous tumor vessel network **(E)**. Magnified image shows single, autofluorescent erythrocytes recorded in the red channel as the source of vascular contrast **(F)**. Segmentation of tumor vessels from *ex vivo* MRI and ultramicroscopy **(G)**. H&E staining of paraffin section, IDH1R132H and CD31 immunohistochemistry **(H)**. MIP: maximum intensity projection. Scale bars are 1 mm in **(A–E,H)**, and 50 μm in **(F)** (200 μm in inset in **B** and 10 μm in inset in **F**).

To investigate the translatability of the MR-UM approach we obtained autopsy material from a patient with a lung adenocarcinoma that had formed several cerebral metastases. We performed MRI of two excised tissue blocks (~2 cm^3^) from the cerebellum and cortex that showed the presence of subcortical metastases within the subcortical white matter (Figures 4A,B). Metastases had a sharp demarcation from the surrounding healthy tissue on MRI and ultramicroscopy (Supplementary Figures 2A,B) and stained positive for cytokeratin AE1/AE3 by immunohistochemistry (Supplementary Figures 2C,D).

### Human Glioblastoma Shows an Invasive Growth Without Clear Separation From the Healthy Parenchyma and Is Highly Angiogenic

We further employed the MR-UM approach to investigate intraoperatively obtained resection material from two glioma patients, one with a glioblastoma (WHO grade IV, *IDH1* wildtype) and one with an oligodendroglioma (WHO grade II, *IDH1* mutant). The glioblastoma showed prominent neoangiogenesis which was already apparent as tubular hypointensities on SWI images acquired before resection (Figure 4C). The high amount of angiogenesis was confirmed by *ex vivo* MRI of the resected specimen (Figure 4D). For correlative ultramicroscopy we relied on endogenous tissue contrast. Interestingly, the pathological blood vessel network of glioblastoma could be visualized by fluorescent, intravascular erythrocytes within tumor blood vessels (Figures 4E,F). Tumor vessels could also be easily segmented both from *ex vivo* MRI based on tubular T2^*^ hypointense structures as well as from form ultramicroscopy data (Figure 4G, Supplementary Movie 4). H&E histology showed the typical morphology of glioblastoma and confirmed multiple pathological neovessels on CD31 immunohistochemistry (Figure 4H).

In contrast, neoangiogenesis was not apparent in an *IDH1* mutant oligodendroglioma specimen. Before resection, MRI showed T2 hyperintense areas but no Gd-contrast enhancement nor intratumoral susceptibility signals (Supplementary Figure 3A). The less aggressive phenotype was also confirmed by *ex vivo* MRI, ultramicroscopy and immunohistochemistry that did not detect pathological neovessels (Supplementary Figures 3B–D).

## Discussion

Glioblastoma is characterized by diffuse tumor infiltration into the adjacent healthy parenchyma and the formation of pathological neovessels. In order to assess these key features, we extended our recently established correlated magnetic resonance and ultramicroscopy (MR-UM) approach to now visualize angiogenesis and tumor invasion concomitantly. We further show as a proof of concept the applicability of our platform to clinical samples. We employed different brain tumor models as well as human glioma and brain metastases samples and find major heterogeneity regarding their degree of neoangiogenesis and growth pattern. MR-UM uses the strength of MRI and ultramicroscopy, namely longitudinal *in vivo* imaging by MRI and the high resolution of ultramicroscopy combined with fluorescent genetic and intravital dye labeling. Additionally, both techniques result in 3D datasets of the entire, intact organ. Spatial information and growth dynamics are important aspects of tumor biology that get lost in the plethora of genetic and epigenetic studies that are currently revolutionizing the field (Jones et al., 2012; Plaisier et al., 2016; Northcott et al., 2017). By applying MR-UM we are able to compare established preclinical models of glioma and brain metastases with the human diseases to determine different biological features of the model. We find that the S24 tumor model showed a highly invasive growth without apparent ongoing neoangiogenesis. In contrast to classical glioma models like Gl261 or U87MG which behave more like brain metastasis, S24 tumors grow slowly and much less angiogenic. Thus, S24 could enable investigations into the infiltrative nature of the disease, a feature that conventional glioma models show only to a limited degree.

We further establish MR-UM on clinical samples. *IDH1* WT glioblastomas display an aggressive behavior with tumor infiltration along white matter structures and the formation of neovessels (Chen et al., 2012). Both aspects can be visualized by MR-UM. We made use of the endogenous contrast of red blood cells to visualize the vascularization, a feature that was also apparent by T2^*^-w MRI. In contrast, brain metastasis in a preclinical model and human metastases specimen showed an encapsulated growth and strong angiogenesis similar to U87MG glioma cells.

Our study builds on existing work that has demonstrated the importance of neoangiogenesis in glioblastoma and its association with overall survival (Birner et al., 2003; Jain et al., 2007; Plate et al., 2012). Also, previous techniques were developed to automatically quantify the microvessel architecture of glioblastoma using fractal-based histopathology (Di Ieva et al., 2011). However, such analyses were restricted to tissue sections, thus loosing the inherent complex 3D-architecture of microvessels. Elegant *in vivo* imaging approaches for vasculature mapping and therapeutic response assessment have involved MR imaging (de Oliveira et al., 2017), optical frequency domain imaging (Vakoc et al., 2009) and intravital microscopy (Farrar et al., 2010; Fukumura et al., 2010). Such techniques are powerful to dissect dynamic processes in the tumor microenvironment and can e.g., delineate treatment effects and elucidate underlying mechanisms. Intravital imaging is however restricted to a depth penetration of ~1 mm below the cortical surface. In contrast optical clearing can assess alterations in the microvasculature and tumor cell invasion in the entire unsectioned brain (Breckwoldt et al., 2016; Lagerweij et al., 2017).

Limitation of our study include the following: human brain tumor samples that are obtained from resections are difficult to register to pre-surgical imaging. This would be possible in stereotactic interventions. However, the tissue geometry and composition changes upon resection. Also, investigations of human samples are not amenable to genetic or intravital dye labeling. Therefore, we relied on endogenous contrast mechanisms such as red blood cells which allowed for delineation of the tumor vasculature. We also tested the recently published iDISCO protocol and proposed antibodies (Renier et al., 2014) for immunohistochemistry to label whole tissue blocks. This would expand possible labels and would be highly desirable. This resulted however in unspecific labeling of the secondary antibody and was difficult to separate from endogenous red blood cell signal (data not shown).

In summary, MR-UM shows the capability to depict hallmarks of brain tumors and brain metastasis in the preclinical and clinical setting. We envision MR-UM as a monitoring platform for the development of drugs targeting tumor invasion and neoangiogenesis. MR-UM can be used for both basic mechanistic and therapeutical studies, thus serving as a tool for the neuro-oncological and neuroscience community.

## Author Contributions

MOB, JB, TK, PW, FW, and BT conceived the experiments. JB, TK, AB, and GS performed tumor injections. MOB, SH, and MH performed MR imaging experiments. MOB, JB, FS, and VV performed histology. FS, AvD, and CH-M processed human material. AH and FK provided analytical tools. MOB, AH, and FK performed data post processing. JB and TK performed UM. MOB and JB analyzed the data. MOB, JB, WW, MP, MB, FW, and BT interpreted the experiments and wrote the paper with input from all co-authors.

### Conflict of Interest Statement

The authors declare that the research was conducted in the absence of any commercial or financial relationships that could be construed as a potential conflict of interest.

## Supplementary Material

Supplementary Figure 1 MRI of a metastasis at the skull base in the A2058 melanoma model (*n* = 5 mice; **A**). Quantification of the tumor volume **(B)** and permeability **(C)**. MRI of a subcortical metastasis over time (early and late stage) **(D)**. T2^*^ after contrast administration and correlated ultramicroscopy image illustrate neovessel formation **(E)**. Scale bars are 1 mm in **(A,B)**, 100 μm in **(C,D)** and 20 μm in insets.

Supplementary Figure 2 *Ex vivo* MRI of a cerebellar metastasis **(A)** and ultramicroscopy **(B)**. H&E staining of paraffin section **(C)** and immunohistochemistry for the cytokeratin marker A1/A3 **(D)**. Scale bars are 2 mm in **(A)** and 200 μm in **(B)**.

Supplementary Figure 3 *In vivo* MRI of human oligodendroglioma (*IDH1* mutant) patient and after specimen resection **(A,B)**. Ultramicroscopy the brain/tumor architecture without apparent tissue distortion or neovascularization. (Auto-) fluorescent signals are caused by red blood cells **(C)**. H&E staining of paraffin section, IDH1R132H and CD31 immunohistochemistry **(D)**. ceT1: Gd-contrast enhanced T1-weighted image. SWI: susceptibility weighted imaging. Scale bars are 1 mm in **(B,D)** and 100 μm in **(C)** and magnified images in **(D)**.

Supplementary Movie 1 Ultramicroscopy of S24-tdTomato tumors shows the extent of tumor infiltration.

Supplementary Movie 2 Segmentation of the corpus callosum showing S24 glioma infiltration.

Supplementary Movie 3 Segmentation of the posterior commissure showing S24 glioma infiltration along the white matter.

Supplementary Movie 4 Segmentation of tumor vessels in a human IDH1 wildtype glioblastoma specimen.

## References

[B1] Alves S. Bode J. Bemelmans A.-P. von Kalle C. Cartier N. Tews B. (2016). Ultramicroscopy as a novel tool to unravel the tropism of AAV gene therapy vectors in the brain. Sci. Rep. 6:28272. 10.1038/srep28272 27320056 PMC4913310

[B2] Birner P. Piribauer M. Fischer I. Gatterbauer B. Marosi C. Ambros P. F.. (2003). Vascular patterns in glioblastoma influence clinical outcome and associate with variable expression of angiogenic proteins: evidence for distinct angiogenic subtypes. Brain Pathol. 13, 133–143. 10.1111/j.1750-3639.2003.tb00013.x 12744467 PMC8095831

[B3] Breckwoldt M. O. Bode J. Kurz F. T. Hoffmann A. Ochs K. Ott M.. (2016). Correlated magnetic resonance imaging and ultramicroscopy (MR-UM) is a tool kit to assess the dynamics of glioma angiogenesis. Elife 5:e11712. 10.7554/eLife.11712 26830460 PMC4755755

[B4] Carmeliet P. Jain R. K. (2011). Molecular mechanisms and clinical applications of angiogenesis. Nature 473, 298–307. 10.1038/nature10144 21593862 PMC4049445

[B5] Chen J. McKay R. M. Parada L. F. (2012). Malignant glioma: lessons from genomics, mouse models, and stem cells. Cell 149, 36–47. 10.1016/j.cell.2012.03.009 22464322 PMC3719882

[B6] de Oliveira E. A. Lazovic J. Guo L. Soto H. Faintuch B. L. Akhtari M.. (2017). Evaluation of magnetonanoparticles conjugated with new angiogenesis peptides in intracranial glioma tumors by MRI. Appl. Biochem. Biotech. 183, 265–279. 10.1007/s12010-017-2443-2 28281182

[B7] Di Ieva A. Grizzi F. Sherif C. Matula C. Tschabitscher M. (2011). Angioarchitectural heterogeneity in human glioblastoma multiforme: a fractal-based histopathological assessment. Microvasc. Res. 81, 222–230. 10.1016/j.mvr.2010.12.006 21192955

[B8] Farrar C. T. Kamoun W. S. Ley C. D. Kim Y. R. Kwon S. J. Dai G.. (2010). *In vivo* validation of MRI vessel caliber index measurement methods with intravital optical microscopy in a U87 mouse brain tumor model. Neuro Oncol. 12, 341–350. 10.1093/neuonc/nop032 20308312 PMC2940602

[B9] Fukumura D. Duda D. G. Munn L. L. Jain R. K. (2010). Tumor microvasculature and microenvironment: novel insights through intravital imaging in pre-clinical models. Microcirculation 17, 206–225. 10.1111/j.1549-8719.2010.00029.x 20374484 PMC2859831

[B10] Furnari F. B. Fenton T. Bachoo R. M. Mukasa A. Stommel J. M. Stegh A.. (2007). Malignant astrocytic glioma: genetics, biology, and paths to treatment. Genes Dev. 21, 2683–2710. 10.1101/gad.1596707 17974913

[B11] Hambardzumyan D. Amankulor N. M. Helmy K. Y. Becher O. J. Holland E. C. (2009). Modeling adult gliomas using RCAS/t-va technology. Transl. Oncol. 2, 89–95. 10.1593/tlo.09100 19412424 PMC2670576

[B12] Haubold C. Schiegg M. Kreshuk A. Berg S. Koethe U. Hamprecht F. A. (2016). Segmenting and tracking multiple dividing targets using ilastik. Adv. Anat. Embryol. Cell Biol. 219, 199–229. 10.1007/978-3-319-28549-8_8 27207368

[B13] Holland E. C. (2001). Gliomagenesis: genetic alterations and mouse models. Nat. Rev. Genet. 2, 120–129. 10.1038/35052535 11253051

[B14] Hyare H. Thust S. Rees J. (2017). Advanced MRI techniques in the monitoring of treatment of gliomas. Curr. Treat. Opt. Neurol. 19:11. 10.1007/s11940-017-0445-6 28349351

[B15] Ishii N. Maier D. Merlo A. Tada M. Sawamura Y. Diserens A. C.. (1999). Frequent co-alterations of TP53, p16/CDKN2A, p14ARF, PTEN tumor suppressor genes in human glioma cell lines. Brain Pathol. 9, 469–479. 10.1111/j.1750-3639.1999.tb00536.x 10416987 PMC8098486

[B16] Jain R. K. di Tomaso E. Duda D. G. Loeffler J. S. Sorensen A. G. Batchelor T. T. (2007). Angiogenesis in brain tumours. Nat. Rev. Neurosci. 8, 610–622. 10.1038/nrn2175 17643088

[B17] Jones D. T. W. Jäger N. Kool M. Zichner T. Hutter B. Sultan M.. (2012). Dissecting the genomic complexity underlying medulloblastoma. Nature 488, 100–105. 10.1038/nature11284 22832583 PMC3662966

[B18] Jung E. Osswald M. Blaes J. Wiestler B. Sahm F. Schmenger T.. (2017). Tweety-homolog 1 drives brain colonization of gliomas. J. Neurosci. 37, 6837–6850. 10.1523/JNEUROSCI.3532-16.2017 28607172 PMC6705725

[B19] Koul D. Shen R. Bergh S. Sheng X. Shishodia S. Lafortune T. A.. (2006). Inhibition of Akt survival pathway by a small-molecule inhibitor in human glioblastoma. Mol. Cancer Ther. 5, 637–644. 10.1158/1535-7163.MCT-05-0453 16546978

[B20] Lagerweij T. Dusoswa S. A. Negrean A. Hendrikx E. M. L. de Vries H. E. Kole J.. (2017). Optical clearing and fluorescence deep-tissue imaging for 3D quantitative analysis of the brain tumor microenvironment. Angiogenesis 20, 533–546. 10.1007/s10456-017-9565-6 28699046 PMC5660146

[B21] Lemke D. Pfenning P.-N. Sahm F. Klein A.-C. Kempf T. Warnken U.. (2012). Costimulatory protein 4IgB7H3 drives the malignant phenotype of glioblastoma by mediating immune escape and invasiveness. Clin. Cancer Res. 18, 105–117. 10.1158/1078-0432.CCR-11-0880 22080438

[B22] Northcott P. A. Buchhalter I. Morrissy A. S. Hovestadt V. Weischenfeldt J. Ehrenberger T.. (2017). The whole-genome landscape of medulloblastoma subtypes. Nature 547, 311–317. 10.1038/nature22973 28726821 PMC5905700

[B23] Osswald M. Blaes J. Liao Y. Solecki G. Gömmel M. Berghoff A. S.. (2016). Impact of blood-brain barrier integrity on tumor growth and therapy response in brain metastases. Clin. Cancer Res. 22, 6078–6087. 10.1158/1078-0432.CCR-16-1327 27521448

[B24] Osswald M. Jung E. Sahm F. Solecki G. Venkataramani V. Blaes J.. (2015). Brain tumour cells interconnect to a functional and resistant network. Nature 528, 93–98. 10.1038/nature16071 26536111

[B25] Park S.-H. Masamoto K. Hendrich K. Kanno I. Kim S.-G. (2008). Imaging brain vasculature with BOLD microscopy: MR detection limits determined by *in vivo* two-photon microscopy. Magn. Reson. Med. 59, 855–865. 10.1002/mrm.21573 18383285 PMC2628751

[B26] Plaisier C. L. O'Brien S. Bernard B. Reynolds S. Simon Z. Toledo C. M.. (2016). Causal mechanistic regulatory network for glioblastoma deciphered using systems genetics network analysis. Cell Syst. 3, 172–186. 10.1016/j.cels.2016.06.006 27426982 PMC5001912

[B27] Plate K. H. Scholz A. Dumont D. J. (2012). Tumor angiogenesis and anti-angiogenic therapy in malignant gliomas revisited. Acta Neuropathol. 124, 763–775. 10.1007/s00401-012-1066-5 23143192 PMC3508273

[B28] Qutaish M. Q. Sullivant K. E. Burden-Gulley S. M. Lu H. Roy D. Wang J.. (2012). Cryo-image analysis of tumor cell migration, invasion, and dispersal in a mouse xenograft model of human glioblastoma multiforme. Mol. Imaging Biol. 14, 572–583. 10.1007/s11307-011-0525-z 22125093 PMC3444683

[B29] Radaelli E. Ceruti R. Patton V. Russo M. Degrassi A. Croci V.. (2009). Immunohistopathological and neuroimaging characterization of murine orthotopic xenograft models of glioblastoma multiforme recapitulating the most salient features of human disease. Histol. Histopathol. 24, 879–891. 10.14670/HH-24.879 19475534

[B30] Renier N. Wu Z. Simon D. J. Yang J. Ariel P. Tessier-Lavigne M. (2014). iDISCO: a simple, rapid methodto immunolabel large tissue samples for volume imaging. Cell 159, 896–910. 10.1016/j.cell.2014.10.010 25417164

[B31] Sahm F. Capper D. Jeibmann A. Habel A. Paulus W. Troost D.. (2012). Addressing diffuse glioma as a systemic brain disease with single-cell analysis. Arch. Neurol. 69, 523–526. 10.1001/archneurol.2011.2910 22158715

[B32] Schmitt M. Pawlita M. (2009). High-throughput detection and multiplex identification of cell contaminations. Nucleic Acids Res. 37, e119. 10.1093/nar/gkp581 19589807 PMC2764421

[B33] Schwarz M. K. Scherbarth A. Sprengel R. Engelhardt J. Theer P. Giese G. (2015). Fluorescent-protein stabilization and high-resolution imaging of cleared, intact mouse brains. PLoS ONE 10:e0124650. 10.1371/journal.pone.0124650 25993380 PMC4439039

[B34] Sherwin S. A. Sliski A. H. Todaro G. J. (1979). Human melanoma cells have both nerve growth factor and nerve growth factor-specific receptors on their cell surfaces. Proc. Natl. Acad. Sci. U.S.A. 76, 1288–1292. 10.1073/pnas.76.3.1288 375235 PMC383236

[B35] Smits M. van den Bent M. J. (2017). Imaging correlates of adult glioma genotypes. Radiology 284, 316–331. 10.1148/radiol.2017151930 28723281

[B36] Vakoc B. J. Lanning R. M. Tyrrell J. A. Padera T. P. Bartlett L. A. Stylianopoulos T.. (2009). Three-dimensional microscopy of the tumor microenvironment *in vivo* using optical frequency domain imaging. Nat. Med. 15, 1219–1223. 10.1038/nm.1971 19749772 PMC2759417

[B37] Wälchli T. Mateos J. M. Weinman O. Babic D. Regli L. Hoerstrup S. P.. (2015). Quantitative assessment of angiogenesis, perfused blood vessels and endothelial tip cells in the postnatal mouse brain. Nat. Protoc. 10, 53–74. 10.1038/nprot.2015.002 25502884

[B38] Weil S. Osswald M. Solecki G. Grosch J. Jung E. Lemke D.. (2017). Tumor microtubes convey resistance to surgical lesions and chemotherapy in gliomas. Neuro Oncol. 19, 1316–1326. 10.1093/neuonc/nox070 28419303 PMC5596180

[B39] Wen P. Y. Kesari S. (2008). Malignant gliomas in adults. NEJM 359, 492–507. 10.1056/NEJMra0708126 18669428

[B40] Wilson D. Roy D. Steyer G. Gargesha M. Stone M. McKinley E. (2008). Whole mouse cryo-imaging. Proc. SPIE Int. Soc. Opt. Eng. 6916, 69161I−69161I9. 10.1117/12.772840 19756215 PMC2743345

[B41] Zhu Z. Khan M. A. Weiler M. Blaes J. Jestaedt L. Geibert M.. (2014). Targeting self-renewal in high-grade brain tumors leads to loss of brain tumor stem cells and prolonged survival. Cell Stem Cell 15, 185–198. 10.1016/j.stem.2014.04.007 24835569

