# Supplementary figures and images for: Correlated MRI and Ultramicroscopy (MR-UM) of Brain Tumors Reveals Vast Heterogeneity of Tumor Infiltration and Neoangiogenesis in Preclinical Models and Human Disease
Source: Front Neurosci. 2019 Jan 10;12:1004. doi: 10.3389/fnins.2018.01004 (PMC6335617; doi:10.3389/fnins.2018.01004)

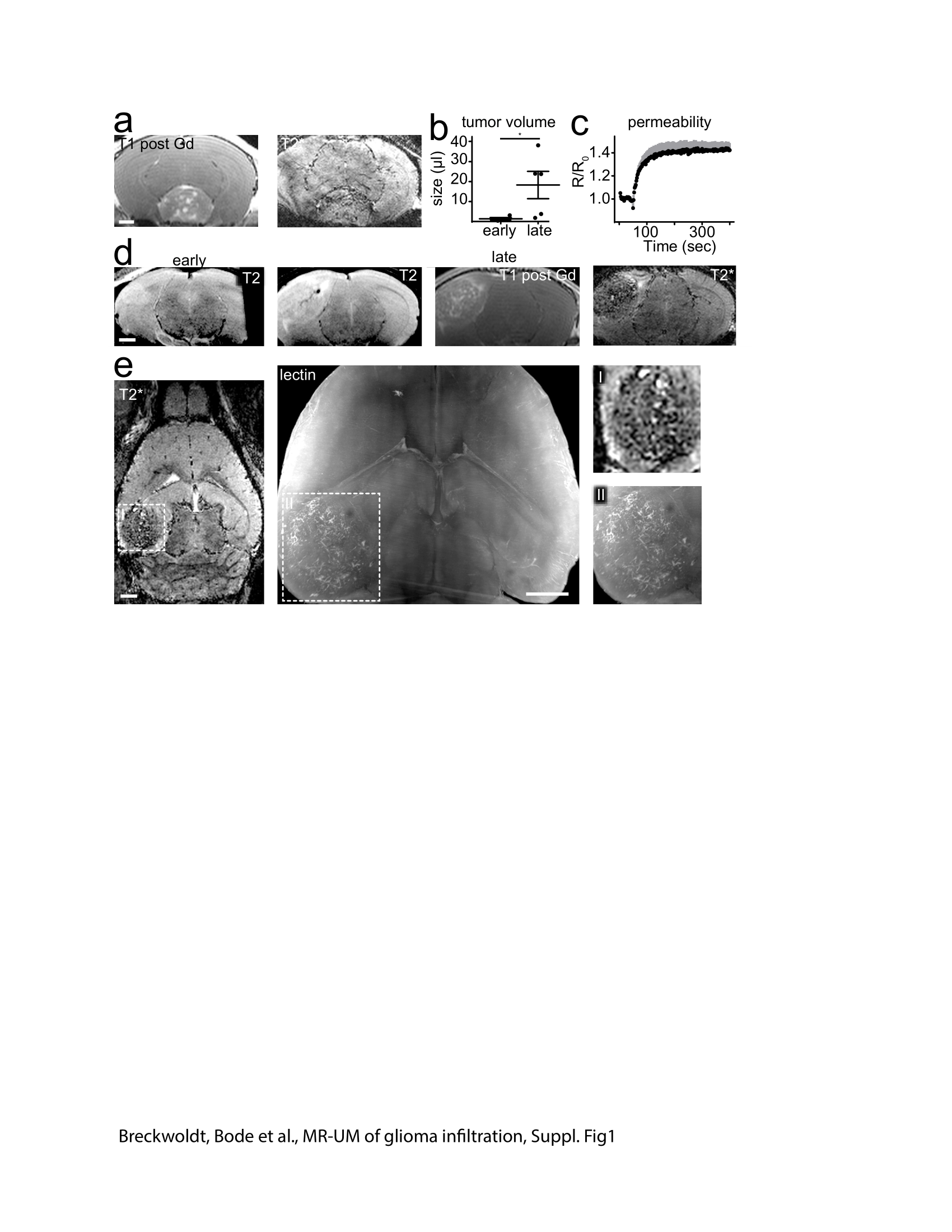

Supplement: Supplementary Figure 1 — MRI of a metastasis at the skull base in the A2058 melanoma model (n = 5 mice; A). Quantification of the tumor volume (B) and permeability (C). MRI of a subcortical metastasis over time (early and late stage) (D). T2* after contrast administration and correlated ultramicroscopy image illustrate neovessel formation (E). Scale bars are 1 mm in (A,B), 100 μm in (C,D) and 20 μm in insets. [file Image_1.TIFF]

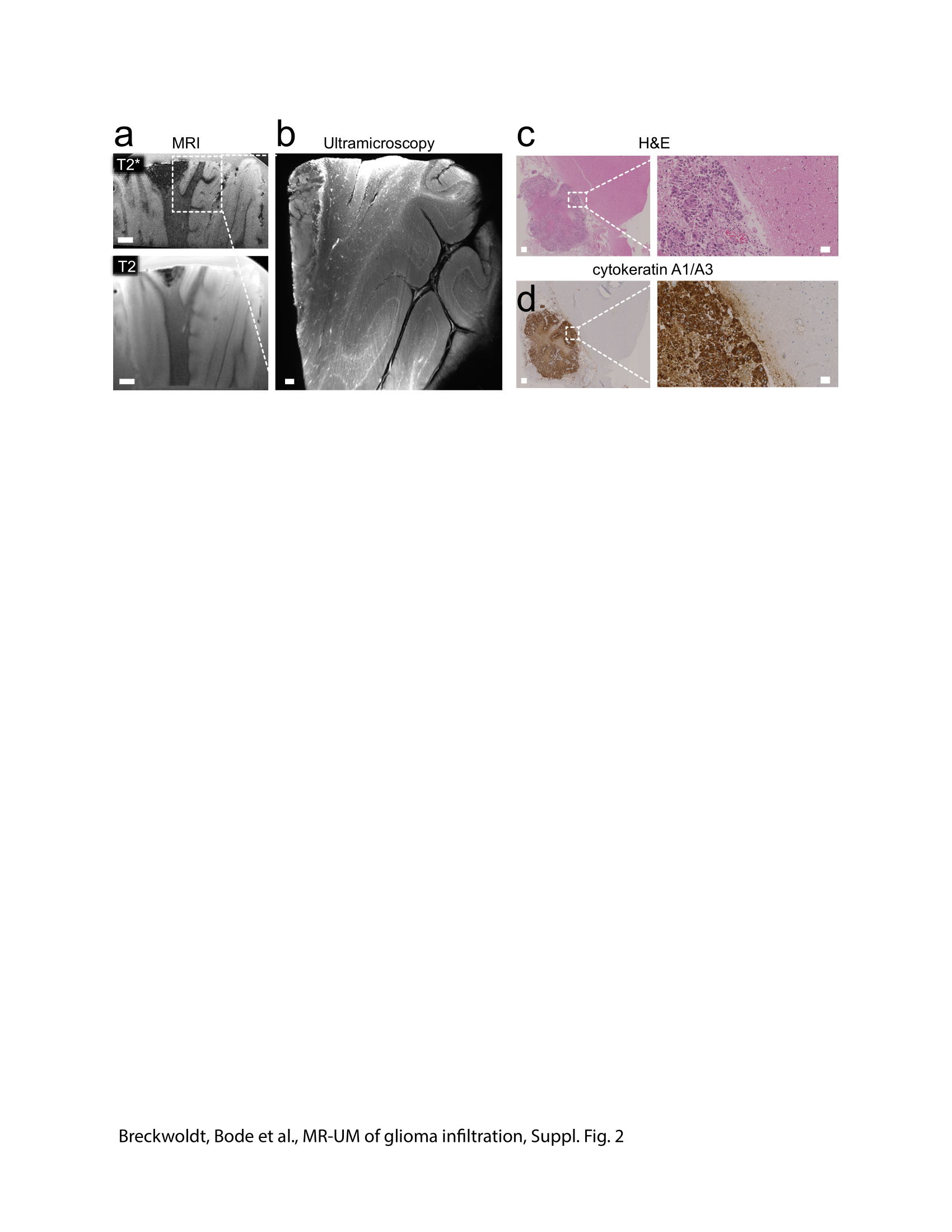

Supplement: Supplementary Figure 2 — Ex vivo MRI of a cerebellar metastasis (A) and ultramicroscopy (B). H&E staining of paraffin section (C) and immunohistochemistry for the cytokeratin marker A1/A3 (D). Scale bars are 2 mm in (A) and 200 μm in (B). [file Image_2.TIFF]

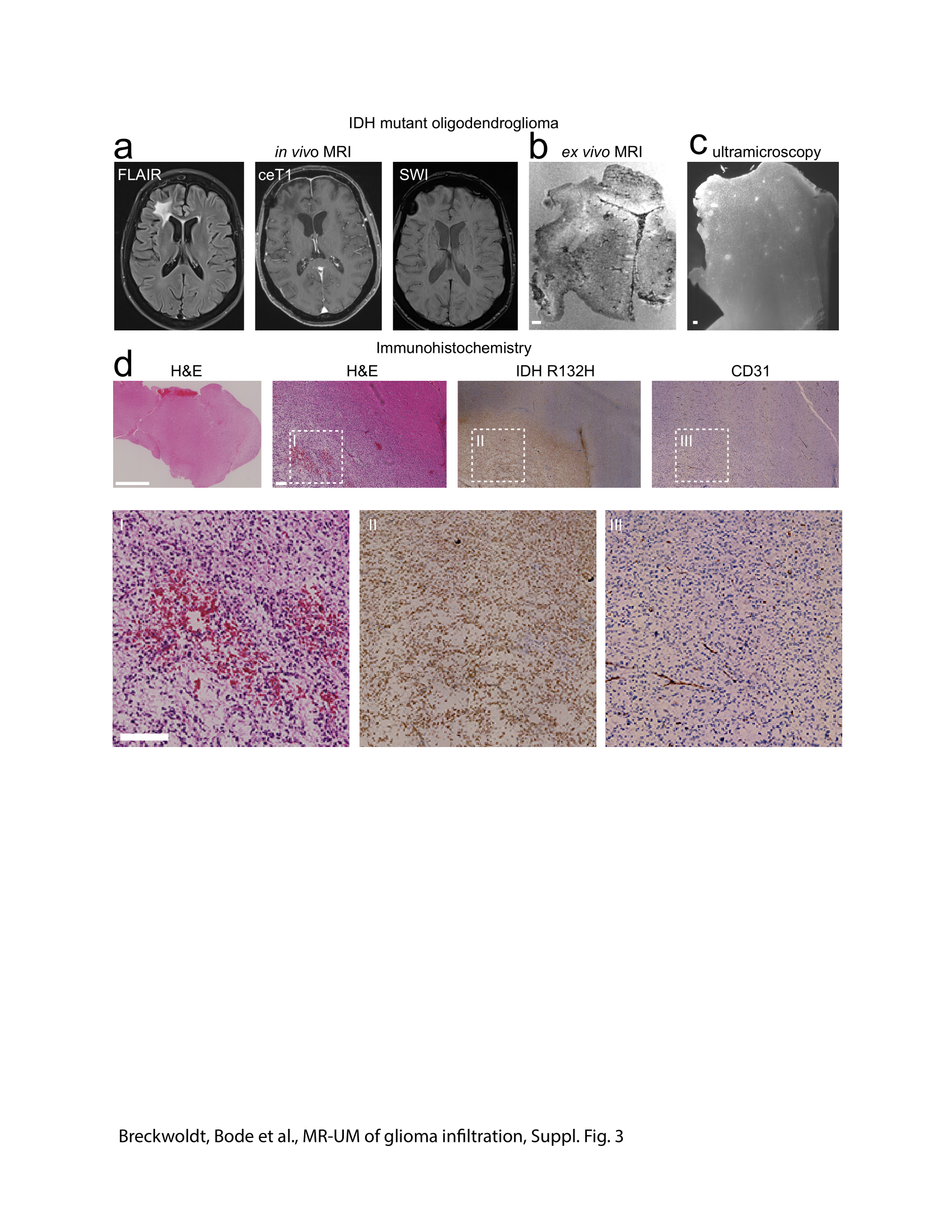

Supplement: Supplementary Figure 3 — In vivo MRI of human oligodendroglioma (IDH1 mutant) patient and after specimen resection (A,B). Ultramicroscopy the brain/tumor architecture without apparent tissue distortion or neovascularization. (Auto-) fluorescent signals are caused by red blood cells (C). H&E staining of paraffin section, IDH1R132H and CD31 immunohistochemistry (D). ceT1: Gd-contrast enhanced T1-weighted image. SWI: susceptibility weighted imaging. Scale bars are 1 mm in (B,D) and 100 μm in (C) and magnified images in (D). [file Image_3.TIFF]
